# Home Respiratory Polygraphy is Useful in the Diagnosis of Childhood Obstructive Sleep Apnea Syndrome

**DOI:** 10.3390/jcm9072067

**Published:** 2020-07-01

**Authors:** Eusebi Chiner, Cristina Cánovas, Virginia Molina, Jose N. Sancho-Chust, Sandra Vañes, Esther Pastor, Miguel Angel Martinez-Garcia

**Affiliations:** 1 Department of Pulmonology, Multidisciplinary Sleep Unit, Sant Joan d′Alacant University Hospital, Ctra Alacant-València s/n, 03550 Sant Joan d′Alacant, Spain; cristinacanovasg@gmail.com (C.C.); virginia_molpe@hotmail.com (V.M.); norbertosancho@gmail.com (J.N.S.-C.); sandravanesbanos@gmail.com (S.V.); epastorespla@gmail.com (E.P.); 2 Department of Pulmonology, Multidisciplinary Sleep Unit, La Fe University and Polytechnic Hospital, 46026 Valencia, Spain; mianmartinezgarcia@gmail.com; 3 Respiratory Diseases Networking Biomedical Research Center (CIBER), Carlos III Health Institute, 28029 Madrid, Spain

**Keywords:** sleep apnea, child, polysomnography, diagnosis, therapy, decision making

## Abstract

The utility of home respiratory polygraphy (HRP) was assessed as an alternative to polysomnography (PSG) in the diagnosis of childhood obstructive sleep apnea syndrome (OSAS). PSG was indicated only in patients with concomitant disease or where HRP results were questionable. The follow-up period was 1 year. We recorded clinical and anthropometric data, physical examination findings, respiratory variables, severity level and choice of therapy. We assessed 121 children, 70 boys and 51 girls, with mean age 7 ± 4 years, mean body mass index (BMI) 19 ± 5 kg/m^2^, and mean BMI percentile 62 ± 38%. We included 104 HRP and 24 PSG recordings. Of the latter, 7 were preceded by HRP (false negatives) and 17 were indicated as the first-choice method owing to concomitant disease. Of the initial HRP recordings, 93% were technically valid. All technically valid HRPs and 96% of PSGs resulted in a diagnosis of OSAS (apnea-hypopnea index 9.5 ± 9.1/h). Thirty-three percent of cases were moderate and 22% severe. Apnea-hypopnea index showed no correlation with BMI or BMI percentile. Adenotonsillectomy was indicated in 93 patients (77%), conservative treatment in 17 (14%), and conservative treatment combined with CPAP/BiPAP in 11 (9%). There were no significant differences between children diagnosed by HRP and by PSG in terms of treatment choice. The prevalence of OSAS in our sample was 96.7%. In conclusion, when the probability of OSAS is high, HRP is usually sufficient for diagnosing the syndrome and establishing therapy in children. PSG is advisable in complex or questionable cases.

## 1. Introduction

Obstructive sleep apnea syndrome (OSAS) causes partial obstruction (hypopnea) or total obstruction (apnea) of the upper airways during sleep, interfering with sleep stages and breathing patterns [1]. Common nocturnal manifestations of OSAS in children are mouth breathing, snoring, enuresis and profuse sweating, while daytime symptoms include hypersomnia, headaches, cognitive and behavioural disorders, poor academic performance, cardiovascular and metabolic disorders, failure to thrive, pulmonary and arterial hypertension and heart failure [2,3,4,5].

Around 2% to 4% of children aged 2 to 6 years have OSAS, and primary snoring (a benign condition that rarely requires treatment) affects between 7% and 16.7% of children aged 6 months to 13 years [6]. The prevalence is similar in children of both sexes. This suggests that sex hormones contribute less to childhood OSAS than to adult OSAS, which predominantly affects men [7]. An increasing rate of childhood obesity helps to explain the persistence of OSAS after adenotonsillectomy (AT) [8,9,10], and improvement after weight loss [11], though obesity is a more influential risk factor in adults. Another predisposing factor is adenotonsillar hypertrophy (AH), which is more frequent in preschoolers (3 to 5 years) [3,8]. Functional factors include neuromuscular diseases and nasal obstruction following viral infections [12,13,14]. Various studies have shown improvements in cognitive disorders after AT [15], probably before age 9, suggesting that the benefits of surgery decrease after this threshold age [16].

Diagnosis is conventionally based on polysomnography (PSG). Though this test has high diagnostic accuracy, it also has high direct and indirect costs [17]. Performing PSG on all children with OSAS would be very expensive, even more so if including children with primary snoring. As a result, and particularly in Europe [10,18], health professionals are looking into simpler and cheaper techniques, such as respiratory polygraphy (RP), which can be done at home (HRP). Clinical guidelines recommend portable monitoring instead of PSG for diagnosis in adults with a high probability of moderate-to-severe OSAS and no comorbidities [19], but make no mention of children. The Spanish consensus document on childhood OSAS suggests performing PSG or hospital RP in children with a high suspicion of the syndrome and with no concomitant diseases [6]. The European Respiratory Society Task Force recommends a stepwise approach to the clinical management of children with suspected OSAS [13]. Recent studies support the use of HRP in children to increase diagnostic efficiency [20]. This technique has some drawbacks, which include underestimation of respiratory events, incapacity to assess neurophysiological parameters, and possible technical problems due to lack of surveillance, but could be useful in moderate-to-severe childhood OSAS. More studies are needed to determine which patients could most benefit from this test [20,21].

Our primary objective was to assess the utility of HRP as a first-choice diagnostic tool in childhood OSAS, and its utility in the therapeutic decision-making process. Our secondary objective was to estimate the prevalence of childhood OSAS in pulmonology clinics.

## 2. Experimental Section

### 2.1. Study Design and Population

We conducted a real-life observational prospective cohort study over 1 year in children who we attended in our pulmonology clinics. We included only children aged 14 years and younger with suspected OSAS, with or without neuropsychic or physical daytime manifestations. The exclusion criteria were refusal to undergo the diagnostic tests and invalid results in both tests.

### 2.2. Clinical Protocol

We collected information from parents/guardians regarding snoring, apnea, nocturnal shortness of breath, restless sleep, nocturnal sweating, enuresis, nasal obstruction, rhinorrhea, mouth breathing, hearing problems, recurring infections, wheezing, heartburn, headaches, hyperactivity or attention deficit, apathy, shyness, drowsiness, poor academic performance, failure to thrive, and other disorders, including poor appetite and polyphagia. We examined the oral cavity to determine the Mallampati score, degree of AH (Brodsky) and palatal width. We also recorded craniofacial morphological characteristics, height, weight, body mass index (BMI) and BMI percentile. In accordance with the growth charts published by the Center for Disease Control and Prevention (CDC) and the American Academy of Pediatrics (AAP), we calculated BMI using the formula weight (kg)/[height (m)]^2^, and categorised the values into age- and sex-specific percentiles. Patients with a BMI above the 95th percentile were considered obese [22].

### 2.3. Diagnostic Protocol

For HRP, patients used a respiratory monitoring device (*Alice PDx, Philips^®^*) that recorded airflow through a nasal cannula, respiratory movements with a chest band, oxygen saturation (SpO2), heart rate and body position. Apnea was defined as >90% interruption of the amplitude of oronasal airflow, with or without desaturation. The definition of hypopnea was a decrease ≥50% in the amplitude of oronasal airflow compared with the pre-event excursion, lasting at least the equivalent of two respiratory cycles, with the reduced amplitude lasting ≥90% of the entire respiratory event compared with the signal amplitude preceding the event, and accompanied by an SpO2 decrease ≥3% [23].

We obtained the apnea–hypopnea index (AHI), baseline SpO2, minimum SpO2, cumulative time of SpO2 below 90% (CT90%), and oxygen desaturation index (ODI) per time in bed (TIB). All the signals were recorded automatically and revised manually by a trained observer. We considered technically valid all HRP recordings lasting at least 3 h and maintaining at least 2 channels, including SpO2 and band or flow [23]. The RP devices were delivered through the patient care centre of the company Home Respiratory Therapies. Parents were shown how to set up the device and resolve problems. After recording, they returned the device for processing. Technically invalid recordings were repeated up to 2 more times. In cases of repeatedly invalid recordings or a possible false negative (AHI < 3/h in HRP and high clinical suspicion), or at the physician′s discretion (concomitant disease, poor cooperation, etc.), PSG was indicated. We used a PSG system (*Sleeplab, Jaeger^®^*) that monitored neurophysiological, respiratory and electrocardiographical variables; body position; snoring; and SpO2 and end-tidal carbon dioxide (EtCO2). PSG recorded the same SpO2 and ODI values as HRP, but adjusted for total sleep time (TST) and taking into account hypopnea associated with desaturation ≥3% and/or arousal [24,25]. The cut-off point for diagnosing OSAS in HRP/PSG was 3 events/hour, and severity was classified as follows: mild (3 to 5), moderate (5 to 10) and severe (>10), according to national and international guidelines [2,6].

### 2.4. Patient Groups

Group A included patients whose final diagnosis and treatment was based on HRP alone, while Group B patients′ final diagnosis and treatment was based on PSG, performed initially or after HRP.

### 2.5. Therapeutic Protocol

After being diagnosed, patients who required AT were referred to the Otorhinolaryngology Department, or to Maxillofacial Surgery where necessary. Conservative treatment consisted of montelukast (4 to 5 mg for at least 6 months) combined with dietary, behavioural and postural modifications. Patients requiring CPAP were shown how to use an automatic CPAP machine at home and were enrolled in an adherence program.

Six to 12 months after treatment, we assessed patients′ progress through HRP or PSG following the initial diagnostic method. Where this was not possible, we actively collected data on patients′ clinical symptoms. We defined *improvement* as >50% decrease in baseline AHI, and *recovery* as return to normal AHI. All patients were followed up for 1 year.

### 2.6. Sample Size Calculation

To compare the 2 groups, assuming a statistical power of 20%, an alpha error of 5% and a loss to follow up rate of 20%, we calculated that we would need 100 patients in our sample.

### 2.7. Statistical Analysis

We presented the data using mean ± standard deviation (SD) for quantitative variables, and absolute values (percentages) for qualitative or dichotomous variables. Using the Kolmogorov-Smirnov test, we determined whether or not the data followed a normal distribution. Where this was the case, we applied the unpaired Student *t* test for independent means and the paired Student *t* test where necessary, for the comparison of 2 groups. For nonnormally distributed variables, we applied the Mann-Whitney test or Wilcoxon signed-rank test, respectively. For qualitative variables we used the chi-square test with Fisher exact text where needed. To compare more than 2 groups we used an ANOVA test with Bonferroni adjustment, except in the case of more than 2 independent groups that did not follow a normal distribution, where we used the Kruskal-Wallis test. To assess correlation, we used Pearson product moment correlation for normally distributed variables and Spearman rank correlation otherwise. For all tests, the significance level was 5%. All data analysis was carried out using the statistical package IBM SPSS Statistics 23.0 *(SPSS, Inc., Chicago, IL, USA).*

### 2.8. Ethical Aspects

We obtained informed consent from parents or guardians to perform the test, and from all patients aged 12 years and older. The project respected the fundamental principles laid down in the Declaration of Helsinki and updated in Edinburgh in the year 2000, and the provisions of Spanish law regarding data protection and bioethics. Our study was approved by the Ethics and Clinical Trials Committee of Sant Joan d′Alacant University Hospital, Alicante, Spain (HUSJ-20-003). This study is registered at the clinical trials registry (www.clinicaltrials.gov; number NCT04340310).

## 3. Results

### 3.1. Overal

**Figure 1:**
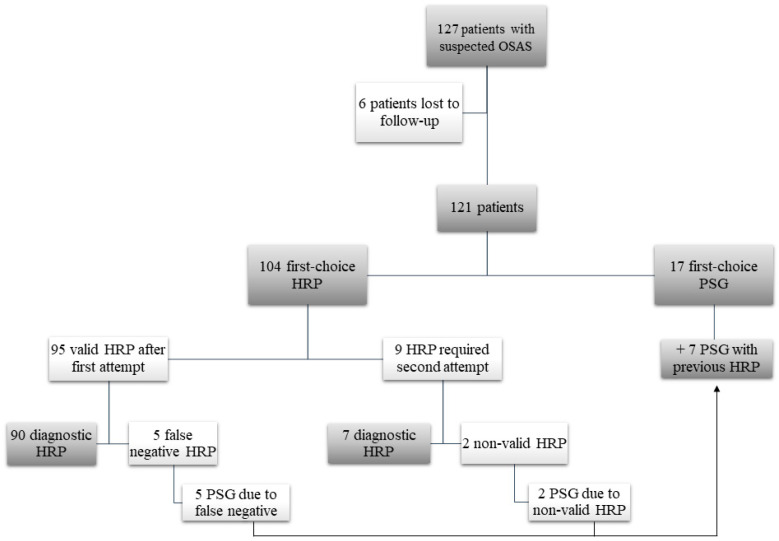
Study flow diagram. OSAS: obstructive sleep apnea syndrome; HRP: home respiratory polygraphy; PSG: polysomnography.

During the study period we assessed 1400 initial hospital visits, of which 162 (11.5%) concerned paediatric patients, and 127 (9%) children with suspected OSAS. Of these 127 patients who met the initial inclusion criteria, 6 were lost to follow up, leaving 121 patients. We assessed recordings from 104 HRPs and 24 PSGs, 7 of which were preceded by HRP. Nine HRPs (11%) had to be repeated after the first attempt was deemed invalid. Two of these second attempts (2.5%) were also invalid, requiring PSG, which led to a diagnosis. The remaining five PSGs with prior HRP were performed because of suspected false negatives. In 17 patients, PSG was indicated from the start at the physician′s discretion (Figure 1).

### 3.2. Study Population

#### 3.2.1. All Patients

We included 70 boys (58%) and 51 girls (42%). Table 1 shows the anthropometric data of the whole sample. The patients were referred from Pulmonology (*n* = 62; 51%), Otorhinolaryngology (*n* = 40; 33%), Paediatrics (*n* = 14; 12%) and other departments (*n* = 5; 4%). Concomitant diseases affected 30 patients (25%), of whom 17 (14%) underwent PSG from the start. These diseases were Down syndrome (*n* = 3), neurofibromatosis (*n* = 2), hearing loss (*n* = 2), lichen striatus, cerebral palsy, pulmonary sequestration, myotonic dystrophy type 1, chromosomal aberration, achondroplasia, migraine, Prader-Willi syndrome, night terrors, and Williams syndrome. The 13 remaining patients with concomitant diseases (asthma, rhinitis, polyposis) underwent HRP. 

**Figure 2:**
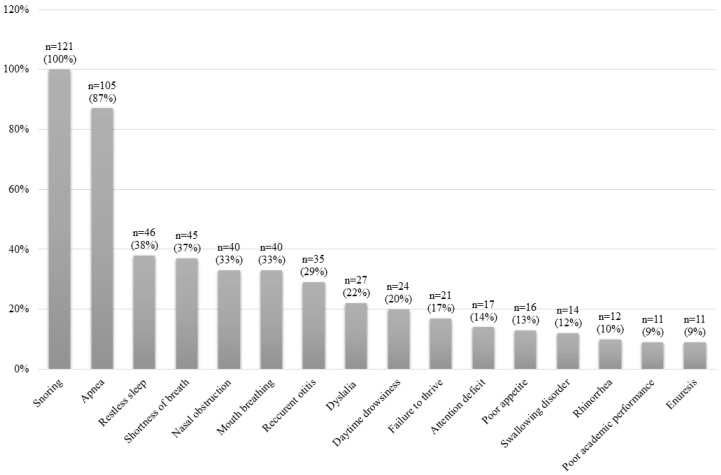
Clinical characteristics for the whole study sample.

**Figure 3:**
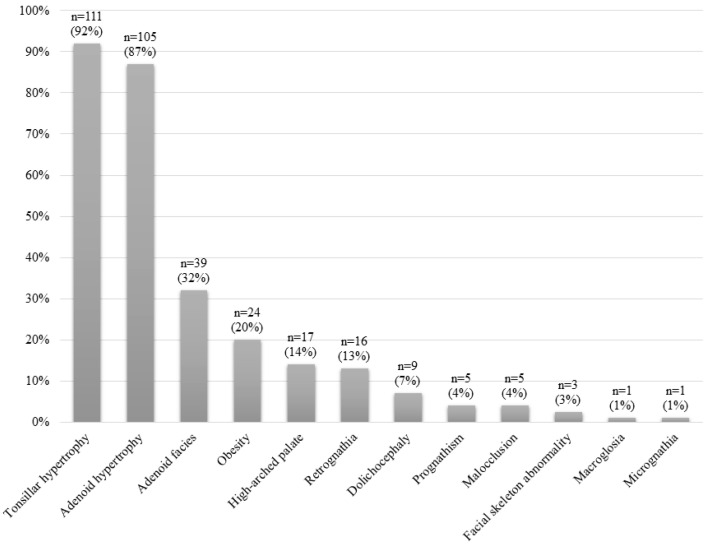
Physical examination findings for the whole study sample.

Figure 2 shows the clinical manifestations of the whole sample. Figure 3 shows the physical examination findings of all patients. The most common findings were AH, adenoid facies, obesity, high-arched palate and retrognathia.

**Figure 4:**
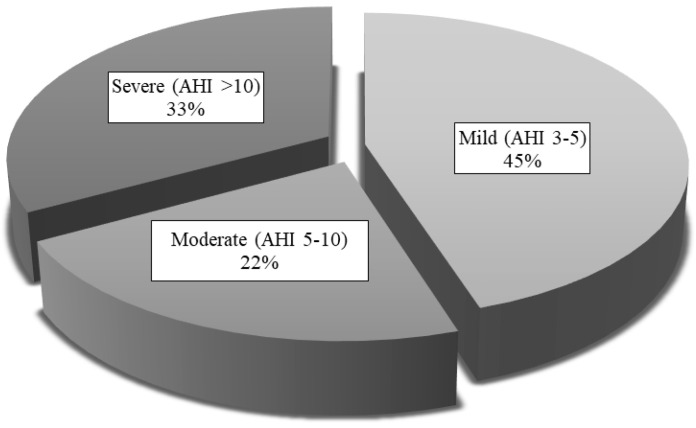
OSAS severity levels for the whole study sample. AHI: apnea-hypopnea index.

Table 2 presents the respiratory variables of all patients, collected using both diagnostic tests. Of the 121 patients, only 4 were considered non-OSAS (3 with AHI < 1/h in HRP and 1 with AHI < 2/h in PSG), meaning the prevalence of OSAS in our sample was 96.7%. Figure 4 shows OSAS severity, according to the AHI obtained in HRP or PSG. Mild OSAS was the most common presentation in our sample.

When we correlated AHI with ODI, SpO2, CT90% and the anthropometric parameters of all the patients, we found no correlation between AHI and BMI or BMI percentile. We found a positive correlation between AHI and ODI (*r* = 0.66, *p* < 0.001) and a negative correlation between weight and baseline SpO2 (*r* = −0.37, *p* < 0.01), minimum SpO2 (*r* = −0.36, *p* < 0.01) and CT90% (*r* = −0.38, *p* < 0.01). We found the same correlations in the 24 patients with obesity.

#### 3.2.2. Comparison between Groups

**Figure 5:**
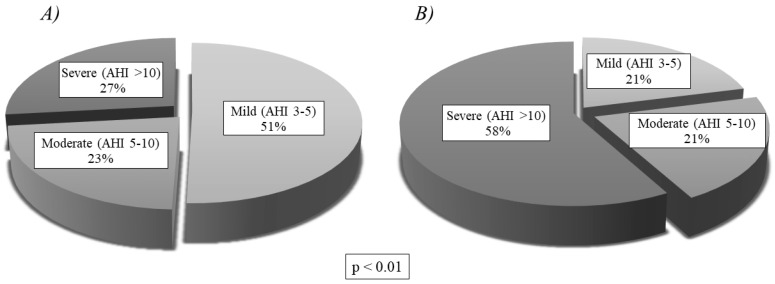
OSAS severity levels by group, showing statistically significant differences (*p* < 0.01): (**A**) Severity of Group A (Home Respiratory Polygraphy); (**B**) Severity of Group B (Polysomnography). AHI: apnea-hypopnea index.

Table 3 compares the clinical manifestations of Group A and Group B. The only significant differences were in concomitant disease (*p* < 0.01) and swallowing disorders (*p* < 0.05), both of which were more common in Group B. Table 4 compares the physical examination findings of the two groups, showing no significant differences. The comparison of anthropometric characteristics and respiratory variables displayed in Table 5 shows statistically significant differences in AHI, ODI and CT90%, which were all more severe in Group B. There are no significant differences in the anthropometric characteristics. Figure 5 expresses severity levels, showing that Group A had a higher proportion of mild OSAS (51%), while Group B had a higher proportion of severe OSAS (58%). Moderate OSAS was similar in both groups. The differences in severity level proportions were statistically significant (*p* < 0.01). 

### 3.3. Treatment

Of the 121 patients, 93 (77%) underwent AT, 17 (14%) received conservative treatment alone (montelukast and/or lifestyle changes), and 11 (9%) received conservative treatment combined with CPAP/BiPAP. There were no significant differences between the groups in terms of treatment choice (Table 6).

### 3.4. Progress

**Figure 6:**
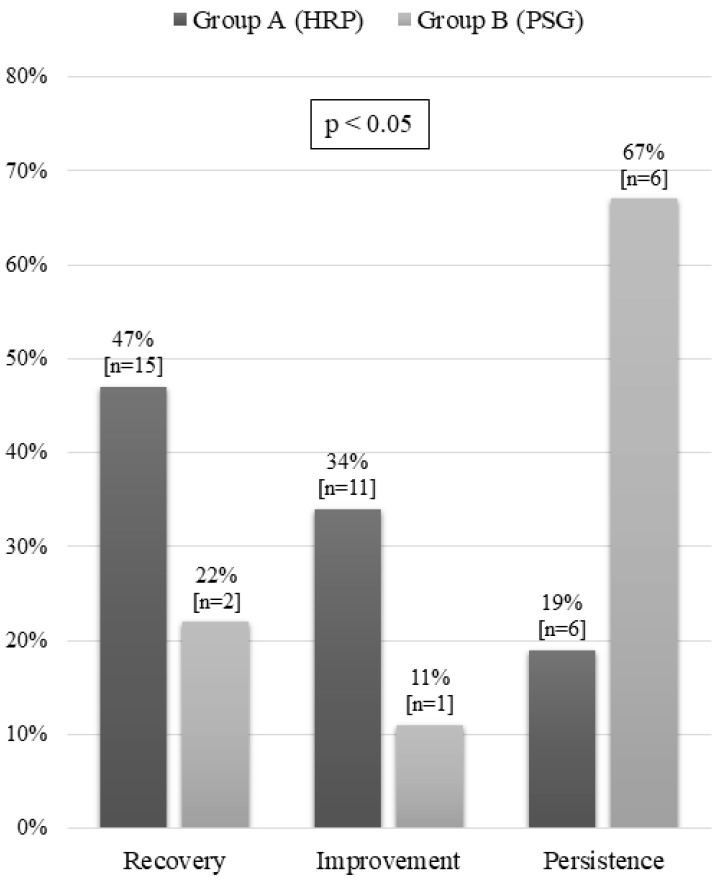
Progress after treatment by group. HRP: home respiratory polygraphy; PSG: polysomnography.

After the therapeutic intervention, we conducted a post-treatment assessment in 41 patients (34% of the total). This assessment involved HRP in 21 patients (50%), a clinical check in 12 (29%) and PSG in 9 (21%). These examinations showed recovery in 17 patients (40%), clinical improvement in 12 (31%), and disease persistence in 12 (29%). There were statistically significant differences in patient progress between the two groups in all three categories. Recovery and improvement were greater in Group A, while persistence of OSAS was more common in Group B (Figure 6).

## 4. Discussion

Childhood OSAS, whether uncomplicated or with severe comorbidities, brings many patients to pulmonology clinics (9% of first visits during the 1-year study period). As there is no clear clinical distinction between OSAS and primary snoring, diagnostic tests are needed to prevent unnecessary surgery [26,27].

In our study, 76% of patients were diagnosed and their treatment established based on HRP alone. We found HRP to be a valid diagnostic method in children, with a similar percentage of technical failures as in the adult population [28]. The test was repeated in only 9% of cases and, according to the methodological criteria, 93% of HRPs were technically valid.

Previous studies have associated AH with OSAS [12]. In our series, other physical features associated with the syndrome were adenoid facies with mouth breathing, micro/retrognathia, dolichocephaly and high-arched palate. Some craniofacial abnormalities and other anatomical factors may be associated with syndromes that affected some of our patients, all of whom had first-choice PSG [2,13,14]. Of the patients with obesity (20% of the total), 87% had AH, meaning obesity was probably not the most significant determining factor. Of note was the lack of correlation between AHI and BMI, unlike in adult OSAS [29]. This correlation was also absent when we analysed only the 24 patients with obesity. This was because only three obese patients had previous AT, meaning AH was the main factor. Paradoxically, OSAS is one of the main causes of failure to thrive, which tends to improve after AT. Childhood obesity is associated with a higher risk of persistent OSAS after AT, with an odds ratio of 3.7 [10].

Gozal et al. compared RP and PSG simultaneously, showing the sensitivity, specificity, positive predictive value (PPV) and negative predictive value (NPV) for different AHI cut-off points. For example, for AHI ≥ 3 events/hour in RP, sensitivity was 70% with a specificity of 100%, PPV of 100% and NPV of 74%. AHI values below three events per hour could be due to a false negative, requiring further diagnostic testing, as in our study [30]. Alonso-Álvarez et al. used HRP in 50 children, finding a sensitivity of 91% and specificity of 94% for AHI > 5.6 events/hour when simultaneously comparing RP and PSG the next day. They concluded that HRP may be useful in children with OSAS [20]. Studies performed by Brockmann et al. (101 children, median age 2.8 years) [31] and Rosen et al. (850 children, aged 8 to 11 years old) [32] in patients with sleep-disordered breathing who underwent RP showed a good positive predictive value of RP, with a sensitivity of 88% and a specificity of 98% for AHI > 5 events/hour [33].

Scalzitti et al. investigated the ability of a portable monitor to diagnose OSAS in children aged 2 to 17 years, analysing sensitivity and specificity compared to PSG [34]. The sensitivity of HRP was best when the device was worn in a sleep laboratory (81.5%), with a specificity of 60%, while the sensitivity was lower (70%), and the specificity was only 42.9% when the monitor was worn at home [34]. The results of that study were probably influenced by the age of the patients, because children under 5 years old had a significantly higher error with the portable device compared to PSG [34]. The AHI measurements from the home sleep test and from the PSG test did not differ significantly in children aged 6 years and older [34]. In paediatric patients, one of the technical challenges is obtaining an adequate nasal airflow signal [35]. Some children with sleep-disordered breathing are mouth breathers, meaning their nasal airflow signal may be reduced [35]. Smaller children may not tolerate the nasal cannula, which will influence RP results. The nasal cannula can also be removed during sleep because of the child′s movements. In one study, Gudnadottir et al. found that most home RP tests were unsuccessful, owing to loss of the nasal flow signal [35]. These problems can be remedied by manual scoring and by establishing, as in our study, a minimum period of recording of 3 h in technically problematic cases, and maintaining at least two channels, including SpO2 and band or flow. However, the more hours of valid recording we obtain, the greater the diagnostic accuracy.

Compared with PSG, RP underestimates AHI because it does not take into account hypopnea associated with microarousal, and it estimates AHI in relation to TIB. Underestimation can be minimised with manual checks and by adjusting the recording time, as in our series. In PSG, moreover, recording time is adjusted for sleep time in view of the possible loss of signal during the test, and the recording is supervised and any problems corrected, resulting in fewer errors than in unsupervised HRP. For this reason, the distribution of severity differed in our HRP and PSG groups, with a higher incidence of mild OSAS in Group A (due to underestimation) and a higher incidence of severe OSAS in Group B (due to higher rate of potentially severe concomitant diseases). Nonetheless, there were no significant differences between the groups in terms of age or anthropometric characteristics, meaning there was no selection bias. Neither were there important differences with regard to symptoms or physical examination findings, and neither test was associated with a particular therapeutic approach: The proportion of patients who received AT, conservative treatment or CPAP/BiPAP was similar in both groups.

One of the most important findings of our study concerns the choice of therapy based on HRP results. We assume that 100% of the PSG tests were useful in the diagnosis and therapeutic decision-making process. HRP combined with PSG helped to determine the best treatment in all cases. Although in our unit PSG is available for more complex or questionable cases, the strategy of starting with HRP even in young patients could be adopted by basic sleep units in coordination with other, larger units that have more resources. The post-treatment assessment conducted in a third of our cases showed a prevalence of residual OSAS of 29%, which is in line with previous findings [36]. We therefore had to establish additional treatment strategies, such as montelukast alone or combined with CPAP/BiPAP [36,37]. The prevalence of residual OSAS was higher in Group B, possibly owing to the higher proportion of severe OSAS in this group.

Our study has some potential limitations. Firstly, as these patients were previously selected and referred to us by other departments with pretest symptoms suggestive of OSAS, almost the whole study sample was diagnosed with and treated for this syndrome. From this we can infer that in cases of high clinical suspicion, HRP may be particularly useful as it reduces the need to perform more invasive and more expensive tests. The diagnosis and choice of therapy was equally valid in most cases diagnosed by HRP or PSG. We tried to make up for these high prevalence limitations by including all patients referred to us, without prior selection. The diagnostic role of RP remains to be established in children with low probability of OSAS, much as in the adult population [28,38].

The mean age of our patients was 7 years, which is slightly higher than in some studies, though identical to that of other published series, such as the Childhood Adenotonsillectomy Trial (CHAT) [15,39,40]. In our opinion this was not due to selection bias, as all patients were analysed systematically, without exclusion, as part of a real-life study. The age difference of patients who undergo AT depends on different factors, such as the level of suspicion, specialists′ awareness of the disease, the action protocol, access to diagnostic resources, and the health system covering patients′ care.

Another limitation was the lack of consensus on the appropriate AHI cut-off points and severity values in RP. More prospective studies are needed to clarify these uncertainties and establish standard cut-off points for RP, since this test may underestimate the AHI, obliging us to raise the cut-off point or determine the point that gives the greatest sensitivity and specificity for a given device [30].

## 5. Conclusions

In conclusion, 80% of our patients were diagnosed and their treatment established based on HRP alone. There is a high demand for childhood OSAS studies, as this syndrome accounted for 9% of first visits to our department in the study period. The main risk factor is upper airway anatomy, even in patients with obesity. PSG should be reserved for special populations or patients with high pretest probability of OSAS and negative (or borderline negative) HRP results.

## Figures and Tables

**Table 1:** Anthropometric data for the whole study sample.

Parameter	Value
Age (years)	7 ± 4
Weight (kg)	28 ± 15
Height (cm)	122 ± 22
BMI (kg/m^2^)	19 ± 5
BMI percentile	62 ± 38

Data are presented as mean ± SD. BMI: body mass index; SD: standard deviation.

**Table 2:** Respiratory variables of the whole study sample.

Parameter	Value
Duration of recording (minutes)	461 ± 95
AHI (h^−1^)	10 ± 9
ODI (h^−1^)	4 ± 7
Baseline SpO2 (%)	97 ± 2
Minimum SpO2 (%)	81 ± 8
CT90% (minutes)	3 ± 5

Data are presented as mean ± SD. AHI: apnea-hypopnea index; CT90%: cumulative time of SpO2 < 90%; ODI: oxygen desaturation index; SpO2: oxygen saturation.

**Table 3:** Clinical and anthropometric characteristics by group.

	Group A (HRP)	Group B (PSG)	*p* Value
Sex			
- Boy - Girl	46% (55) 35% (42)	12% (15) 7% (9)	NS
Concomitant disease	20% (19)	46% (11)	*p* < 0.001
Snoring	100% (97)	100% (24)	NS
Apnea	87% (84)	88% (21)	NS
Shortness of breath	35% (34)	46% (11)	NS
Restless sleep	36% (35)	46% (11)	NS
Rhinorrhea	12% (12)	0% (0)	NS
Nasal obstruction	32% (31)	38% (9)	NS
Mouth breathing	32% (31)	38% (9)	NS
Tonsillitis	53% (51)	54% (13)	NS
Vomiting/nausea	3% (3)	0% (0)	NS
Swallowing disorder	8% (8)	25% (6)	*p* < 0.05
Night sweats	3% (3)	4% (1)	NS
Hearing problems	5% (5)	8% (2)	NS
Daytime drowsiness	20% (19)	21% (5)	NS
Poor appetite	13% (13)	13% (3)	NS
Frequent otitis media	28% (27)	33% (8)	NS
Shyness	4% (4)	0% (0)	NS
Attention deficit	14% (14)	13% (3)	NS
Poor academic performance	8% (8)	13% (3)	NS
Enuresis	9% (9)	8% (2)	NS
Headaches	3% (3)	4% (1)	NS
Heartburn	1% (1)	0% (0)	NS
Wheezing	8% (8)	13% (3)	NS
Failure to thrive	18% (17)	17% (4)	NS

Data are presented as percentage of patients (number of patients). HRP: home respiratory polygraphy; NS: non-significant; PSG: polysomnography.

**Table 4:** Physical examination findings by group.

	Group A (HRP)	Group B (PSG)	*p* Value
Tonsillar hypertrophy	93% (90)	88% (21)	NS
Adenoid hypertrophy	87% (84)	88% (21)	NS
High-arched palate	14% (14)	13% (3)	NS
Macroglossia	1% (1)	0% (0)	NS
Facial skeleton abnormality	3% (3)	0% (0)	NS
Micrognathia	1% (1)	0% (0)	NS
Prognathism	3% (3)	8% (2)	NS
Retrognathia	16% (15)	4% (1)	NS
Malocclusion	3% (3)	8% (2)	NS
Dolichocephaly	7% (7)	8% (2)	NS
Adenoid facies	34% (33)	25% (6)	NS
Obesity	20% (19)	21% (5)	NS

Data are presented as percentage of patients (number of patients). HRP: home respiratory polygraphy; NS: non-significant; PSG: polysomnography.

**Table 5:** Anthropometric characteristics and respiratory variables by group.

	Group A (HRP) (Mean ± SD)	Group B (PSG) (Mean ± SD)	*p* Value
Age (years)	7 ± 4	7 ± 4	NS
Weight (kg)	28 ± 16	25 ± 11	NS
Height (cm)	123 ± 22	118 ± 20	NS
BMI (kg/m^2^)	18 ± 4	20 ± 5	NS
BMI percentile	60 ± 40	72 ± 32	NS
Recording duration (mins)	473 ± 92	411 ± 90	NS
AHI (h^−1^)	8 ± 8	14 ± 11	*p* < 0.01
ODI (h^−1^)	3 ± 5	8 ± 10	*p* < 0.001
Baseline SpO2 (%)	97 ± 1	97 ± 1	NS
Minimum SpO2 (%)	80 ± 8	83 ± 9	NS
CT90%	3 ± 4	6 ± 8	*p* < 0.01

Data are presented as mean ± SD. AHI: apnea-hypopnea index; BMI: body mass index; CT90%: Cumulative time of oxygen saturation <90%; HRP: home respiratory polygraphy; ODI: oxygen desaturation index; NS: non-significant; PSG: polysomnography.

**Table 6:** Therapeutic approach by group.

	Group A (HRP) % (n)	Group B (PSG) % (n)	*p* Value
Adenotonsillectomy	76% (74)	79% (19)	NS
Conservative	17% (16)	4% (1)	NS
CPAP	7% (7)	13% (3)	NS
BiPAP	0% (0)	4% (1)	NS

Data are presented as percentage of patients (number of patients). BiPAP: bilevel positive airway pressure; CPAP: continuous positive airway pressure; HRP: home respiratory polygraphy; NS: non-significant; PSG: polysomnography.

## References

[1] American Thoracic Society (1996). Standards and indications for cardiopulmonary sleep studies in children. Am. J. Respir. Crit. Care Med..

[2] Marcus C.L., Brooks L.J., Draper K.A., Gozal D., Halbower A.C., Jones J., Schechter M.S., Ward S.D., Sheldon S.H., Shiffman R.N. (2012). Diagnosis and management of childhood obstructive sleep apnea syndrome. Pediatrics.

[3] Gómez-Pastrana D., Álvarez Gil D. (2017). Síndrome de apneas-hipopneas durante el sueño. Protoc. Diagn. Ter. Pediatr..

[4] Gusmao T.S., Pompéia S., Miranda M.C. (2018). Cognitive and behavioral effects of obstructive sleep apnea syndrome in children: A systematic literature review. Sleep Med..

[5] Trosman I., Trosman S.J. (2017). Cognitive and behavioral consequences of sleep disordered breathing in children. Med. Sci..

[6] Alonso-Álvarez M.L., Canet T., Cubell-Alarco M., Estivill E., Fernández-Julian E., Gozal D., Jurado-Luque M.J., Lluch-Roselló M.A., Martínez-Pérez F., Merino-Andreu M. (2011). Consensus document on sleep apnea-hypopnea syndrome in children (full version). Sociedad Española de Sueño. El Área de Sueño de la Sociedad Española de Neumología y Cirugía Torácica (SEPAR). Arch. Bronconeumol..

[7] Bixler E.O., Vgontzas A.N., Lin H.M., Liao D., Calhoun S., Vela-Bueno A., Fedok F., Vlasic V., Graff G. (2009). Sleep disordered breathing in children in a general population sample: Prevalence and risk factors. Sleep.

[8] Huang Y.S., Guillerminault C. (2017). Pediatric obstructive sleep apnea: Where do we stand?. Adv. Otorhinolaryngol..

[9] Verhulst S.L., Franckx H., Van Gaal L., De Backer W., Desager K. (2009). The effect of weight loss on sleep-disordered breathing in obese teenagers. Obesity.

[10] O’Brien L.M., Sitha S., Baur L.A., Waters K.A. (2006). Obesity increases the risk for persisting obstructive sleep apnea after treatment in children. Int. J. Pediatr. Otorhinolaryngol..

[11] Andersen I.G., Holm J.C., Homoe P. (2016). Obstructive sleep apnea in obese children and adolescents, treatment methods and outcome of treatment—A systematic review. Int. J. Pediatr. Otorhinolaryngol..

[12] Al Ali A., Richmond S., Popat H., Playle R., Pickles T., Zhurov A.I., Marshall D., Rosin P.L., Henderson J., Bonuck K. (2015). The influence of snoring, mouth breathing and apnoea on facial morphology in late childhood: A three-dimensinal study. BMJ Open.

[13] Kaditis A.G., Alonso-Álvarez M.L., Boudewyns A., Alexopoulos E.I., Ersu R., Joostes K., Larramona H., Miano S., Narang I., Trang H. (2016). Obstructive sleep disordered breathing in 2- to 18-year-old children: Diagnosis and management. Eur. Respir. J..

[14] Reckley L.K., Fernandez-Salvador C., Camacho M. (2018). The effect of tonsillectomy on obstructive sleep apnea: An overview of systematic reviews. Nat. Sci. Sleep.

[15] Marcus C.L., Moore R.H., Rosen C.L., Giordani B., Garetz S.L., Taylor H.G., Mitchell R.B., Amin R., Katz E.S., Arens R. (2013). A randomized trial of adenotonsillectomy for childhood sleep apnea. N. Engl. J. Med..

[16] De Miguel-Díez J., Villa-Asensi J.R., Álvarez-Sala J.L. (2003). Prevalence of sleep-disordered breathing in children with down syndrome: Polygraphic findings in 108 children. Sleep.

[17] Chiner E. (2001). Approach to the cost of polysomnography In a Spanish hospital. Internet J. Pulm. Med..

[18] Lumeng J.C., Chervin R.D. (2008). Epidemiology of pediatric obstructive sleep apnea. Proc. Am. Thorac. Soc..

[19] Collop N.A., Anderson W.M., Boehlecke B., Claman D., Goldberg R., Gottlieb D.J., Hudgel D., Sateia M., Schwab R. (2007). Clinical guidelines for the use of unattended portable monitors in the diagnosis of obstructive sleep apnea in adult patients. Portable monitoring task force of the American academy of sleep medicine. J. Clin. Sleep Med..

[20] Alonso-Álvarez M.L., Terán-Santos J., Ordax-Carbajo E., Cordero-Guevara J.A., Navazo-Egüia A.I., Kheirandish-Gozal L., Gozal D. (2015). Reliability of home respiratory polygraphy for the diagnosis of sleep apnea in children. Chest.

[21] Sardón Prado O., González Pérez-Yarza E., Aldasoro Ruiz A., Estévez Domingo M., Mintegui Aranburua J., Korta Murua J., Emparanza Knörrb J.L. (2006). Rentabilidad de la poligrafía respiratoria del sueño realizada en el domicilio. Anales de Pediatría.

[22] WHO Growth Reference 5–19 years. http://www.who.int/growthref/en/.

[23] Masa J.F., Corral J., Pereira R., Duran-Cantolla J., Cabello M., Hernández-Blasco L., Monasterio C., Alonso A., Chiner E., Rubio M. (2011). Effectiveness of home respiratory polygraphy for the diagnosis of sleep apnoea and hypopnoea syndrome. Thorax.

[24] Rechtschaffen A., Kales A. (1968). A Manual of Standardized Terminology, Techniques and Scoring System of Sleep Stages in Human Subjects.

[25] Moser D., Anderer P., Gruber G., Parapatics S., Loretz E., Boeck M., Kloesch G., Heller E., Schmidt A., Danker-Hopfe H. (2009). Sleep classification according to AASM and rechtschaffen & kales: Effects on sleep scoring parameters. Sleep.

[26] Nieminen P., Tolonen U., Lopponen H. (2000). Snoring and obstructive sleep apnea in children. Arch. Otolaryngol. Head Neck Surg..

[27] Carroll J.L., McColley S.A., Marcus C.L., Curtis S., Loughlin G.M. (1995). Inability of clinical history to distinguish primary snoring from obstructive sleep apnea syndrome in children. Chest.

[28] Masa J.F., Corral J., Pereira R., Duran-Cantolla J., Cabello M., Hernández-Blasco L., Monasterio C., Alonso-Fernandez A., Chiner E., Vázquez-Polo F.J. (2013). Effectiveness of sequential automatic-manual home respiratory polygraphy scoring. Eur. Respir. J..

[29] Lloberes P., Durán-Cantolla J., Martínez-García M.A., Marín J.M., Ferrer A., Corral J., Masa J.F., Parra O., Alonso-Álvarez M.L., Terán-Santos J. (2011). Diagnóstico y tratamiento del síndrome de apneas-hipopneas del sueño. Normativa SEPAR. Arch. Bronconeumol..

[30] Tan H.L., Gozal D., Ramírez H.M., Bandla H.P., Kheirandish-Gozal L. (2014). Overnight polysomnography versus respiratory polygraphy in the diagnosis of pediatric obstructive sleep apnea. Sleep.

[31] Brockmann P.E., Perez J.L., Moya A. (2013). Feasibility of unattended home polysomnography in children with sleep-disordered- breathing. Int. J. Pediatr. Otorhinolaryngol..

[32] Rosen C.L., Larkin E.K., Kirchner H.L., Emancipator J.L., Bivins S.F., Surovec S.A., Martin R.J., Redline S. (2003). Prevalence and risk factors for sleep-disordered breathing in 8 to 11 year-old children: Association with race and prematurity. J. Pediatr..

[33] Franco P., Bourdin H., Braun F., Briffod J., Pin I., Challamel M.J. (2017). Overnight polysomnography versus respiratory polygraphy in the diagnosis of pediatric obstructive sleep apnea. Arch. Pediatr..

[34] Scalzitti N., Hansen S., Maturo S., Lospinoso J., O’Connor P. (2017). Comparison of home sleep apnea testing versus laboratory polysomnography for the diagnosis of obstructive sleep apnea in children. Int. J. Pediatr. Otorhinolaryngol..

[35] Gudnadottir G., Hafsten L., Redfors S., Ellegård E., Hellgren J. (2019). Respiratory polygraphy in children with sleep-disordered breathing. J. Sleep Res..

[36] Imanguli M., Ulualp S.O. (2016). Risk factors for residual obstructive sleep apnea after adenotonsillectomy in children. Laryngoscope.

[37] Gozal D., Tan H.L., Kheirandish-Gozal L. (2020). Treatment of obstructive sleep apnea in children: Handling the unknown with precision. J. Clin. Med..

[38] Tan H.L., Kheirandish-Gozal L., Gozal D. (2015). Pediatric home sleep apnea testing; slowly getting there!. Chest.

[39] Liu C.C., Chaput K.H., Kirk V., Yunker W. (2019). Overnight oximetry in children undergoing adenotonsillectomy: A single center experience. J. Otolaryngol. Head Neck Surg..

[40] Weinstock T.G., Rosen C.L., Marcus C.L., Garetz S., Mitchell R.B., Amin R., Paruthi S., Katz E., Arens M., Weng J. (2014). Predictors of obstructive sleep apnea severity in adenotonsillectomy candidates. Sleep.

